# Non-contrast computed tomography characteristics in a large cohort of cystinuria patients

**DOI:** 10.1007/s00345-020-03509-0

**Published:** 2020-11-09

**Authors:** Hannah Warren, Daniel Poon, Rohit Srinivasan, Kerushan Thomas, Giles Rottenberg, Matthew Bultitude, Kay Thomas

**Affiliations:** 1 Department of Urology, Guy’s and St Thomas’ NHS Foundation Trust, London, UK; 2 Department of Radiology, Guy’s and St Thomas’ NHS Foundation Trust, London, UK; 3 King’s College London, London, UK

**Keywords:** Cystine, Cystinuria, Hounsfield units, Attenuation, CT

## Abstract

**Purpose:**

Cystine stones are widely considered hard and difficult to treat. Hounsfield Units (HU) are used in other stone types to estimate ‘hardness’ and treatments based on that finding. Our objective was to report mean HU of cystine stones in vivo in a large case series of cystinuria patients and assess for differences in genotype.

**Methods:**

A prospective case series of cystinuria patients referred to a specialist centre was analysed. CT imaging was assessed by two independent radiologists to determine in vivo attenuation of cystine calculi. Mean HU was compared for both cystinuria genes (SLC3A1 and SLC7A9) using an independent t-test.

**Results:**

164 adult cystinuric patients were identified (55% male), median age 43 years (range 18–80). Median follow up was 31 months (IQR 10–62). Genetic data available for 153/164 (93%) demonstrated 97 SLC3A1 (63%) and 55 (36%) SLC7A9 mutations (39 homozygous, 16 heterozygous) and one heterozygous for both SLC3A1/SLC7A9. 107 patients had CT images available demonstrating calculi. Median HU across the cohort was 633 (5th to 95th centile 328–780). There was no difference in mean HU between SLC3A1 and SLC7A9 genotypes (*p* = 0.68) or homo and heterozygous SLC7A9 (*p* = 0.70). Mean HU correlated with stone size (Pearson correlation coefficient = 0.51, *p* < 0.001).

**Conclusion:**

In this large single centre cystinuria cohort, mean HU was low for stones that are difficult to treat. Calculi of < 800 HU should prompt consideration of a cystinuria diagnosis. Attenuation was not associated with genotype, and distinct ‘smooth’ and ‘rough’ stones were not observed. Calculi with HU > 1000 are unlikely pure cystine, and in a known cystinuric would suggest conversion to another stone type.

**Electronic supplementary material:**

The online version of this article (10.1007/s00345-020-03509-0) contains supplementary material, which is available to authorized users.

## Introduction

Cystinuria is a phenotypically heterogeneous condition in which defective renal tubular transport leads to supersaturation and crystallisation of the amino acid cystine in the urinary collecting system. It is not known how different gene mutations affect the degree of protein dysfunction and how this translates to clinical phenotype. Recurrent calculi and multiple treatment episodes characterise cystinuria, with stones that are considered hard and relatively treatment resistant [1].

We have previously reported 57 mutations in one of two genes (SLC3A1 and SLC7A9) in a large UK cohort of cystinuria patients [2]. We have modelled the degree of protein dysfunction and its association with disease severity [3], and in sibling studies suggested that patient genotype alone does not determine phenotype [4].

In vitro and in vivo studies have assessed the utility of computed tomography attenuation in predicting stone composition [5, 6], however the standard of care remains analysis with X-ray diffraction and infra-red spectroscopy following extraction or spontaneous passage. In contemporary urological practice, attenuation measured in mean Hounsfield Units (HU) on non-contrast computed tomography (NCCT) is used to judge stone hardness, and treatment based on this finding [7, 8]. High stone attenuation value on NCCT (> 1000 HU) has been shown to be a predictor of treatment failure with shockwave lithotripsy and alternative treatments are advised [8]. In cystinuria, the internal structure of a calculus has been shown to be associated with susceptibility to treatment by extracorporeal shockwave lithotripsy (ESWL) [9]. ‘Rough’ cystine stones with internal voids on micro CT have demonstrated lower attenuation values compared to ‘smooth’ homogenous stones on helical CT in vitro [9]. It is not known what causes this difference in structure, and if it is related to the responsible cystinuria gene.

Non-invasive determination of stone composition and hardness would allow treatment options to be better selected for the individual’s stone, and treatments that are unlikely to be successful could be avoided along with their associated risks.

Cystinuria is a rare condition and previous studies evaluating stones of this type have included only small numbers. The primary purpose of this study was to report NCCT attenuation in HU of stones from a large database of cystinuric patients in a real-world setting, with the secondary aim of assessing if cystinuria gene was associated with the attenuation of the stone.

## Methods

A prospective series of consecutive adult patients (> 18 years old) referred with a diagnosis of cystinuria to a specialist service between 2008 and 2018 was reviewed. The observational nature of this study meant it was exempt from ethical approval requirement, in accordance with guidance from the UK National Health Service Health Research Authority. All patients attending this clinic are offered genetic testing using previously described methodology [2] and regular follow up to facilitate stone prevention and management [10]. Demographic and follow up data were collected from electronic patient records.

Radiology records for all patients were reviewed to identify NCCT imaging confirming the presence of urinary tract calculi. In individuals with multiple independent stone episodes captured on NCCT, we reviewed the scan with the greatest stone burden by volume.

NCCT imaging was independently assessed by two radiologists according to a pre-agreed protocol. When multiple calculi were present, measurements were taken from the largest stone. The largest stone diameter in any dimension was recorded. The mean attenuation value was measured in HU by drawing a circular region of interest at the centre of the stone at its largest diameter using bone windows and axial slices. Mean HU across the circular region of interested was recorded in keeping with techniques described in the literature [9, 11]. We collected energy settings and slice width data from each NCCT.

Inter-rater reliability was assessed using Cronbach’s alpha co-efficient. We compared mean HU for both cystinuria genes (SLC3A1 and SLC7A9) using an independent t-test. The Pearson correlation coefficient was used to assess association between stone size and measured HU. STATA v 15.1 was used for all analyses, and *p* value < 0.05 was considered significant.

## Results

Electronic records of 164 adult cystinuric patients were reviewed. Median follow up time was 31 months (IQR 10–62). The cohort demographic was 55% male: 45% female, with a median age of 43 years (range 18–80). Genetic analysis, available for 153/164 (93%) of patients demonstrated 97 (63%) SLC3A1 mutations and 55 (36%) SLC7A9 mutations [39 homozygous, 16 heterozygous] and one heterozygous for both SLC3A1 and SLC7A9.

NCCT available for review demonstrated urinary tract calculi in 107/164 patients. NCCTs were available from single-energy scanners of different models, from within our own institution and referring centres. CT settings varied between patients. Median energy was 120 kV (interquartile range 120–120), and median slice width 2 mm (IQR 2–2.75 mm).

The median size of patients’ largest stone was 15.7 mm (interquartile range 9.4–28.1 mm). Median HU measurement across the cohort was 633 (5th to 95th centile 328—780). Inter-rater reliability of measurements was excellent for stone diameter (Cronbach’s alpha = 0.98) and good for mean HU (Cronbach’s alpha = 0.85). There was no difference in mean HU between SLC3A1 and SLC7A9 genotypes (*p* = 0.68) or between homo and heterozygous SLC7A9 (*p* = 0.70).

**Fig. 1:**
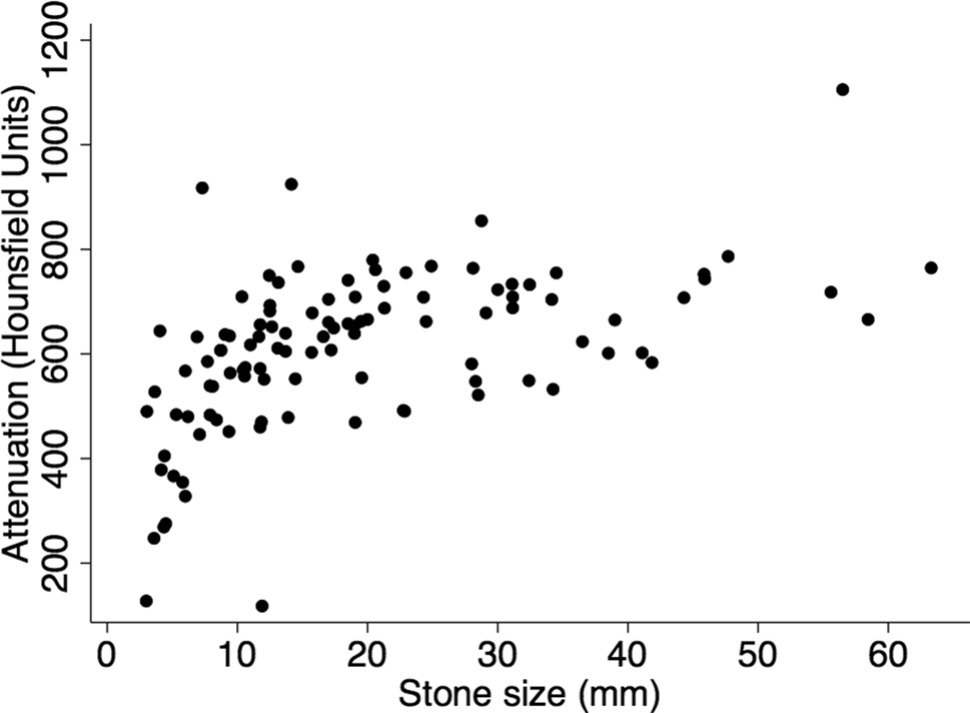
Correlation between stone size and measured mean attenuation. Pearson correlation coefficient 0.51, *p* < 0.001

Attenuation in HU positively correlated with maximum stone diameter, demonstrated in Fig. 1 (Pearson correlation coefficient 0.51, *p* < 0.001).

## Discussion

NCCT is the gold standard imaging modality for the diagnosis of ureteric colic and is recommended in planning access for percutaneous nephrolithotomy [12]. Information from NCCT imaging including stone size, position and attenuation is used to help guide treatment. This study is the largest reported series of cystinuria patients and has demonstrated that the vast majority of cystine stones have HU < 800. We advocate considering a diagnosis of cystinuria when evaluating a patient with a calculus of HU < 800 on NCCT, particularly if of young age or with recurrent episodes.

Attenuation of calculi on NCCT does not appear to be associated with the causative cystinuria gene, adding further to the evidence that phenotype may be influenced by complex genetic and environmental interactions.

The major strength of the current study is the large number of cystinuria patients, accumulated over a decade of experience running a specialist cystinuria clinic. Radiographic assessment by two independent radiologists with good agreement demonstrates the reproducibility of mean HU measurement.

There has been much interest in determining stone composition of all types by NCCT characteristics to allow those with relatively ESWL-resistant composition to avoid the potential complications associated with this treatment when they are unlikely to derive benefit. Recent work by Bonatti et al. shows promise of dual energy CT in determining the chemical composition of urinary tract calculi, by assessing the attenuation ratio of the stones from dual energy images, when compared to single energy CT [13]. The work showed improved sensitivity and specificity in distinguishing uric acid from cystine and calcium calculi. This builds on work by Boll et al. that showed post-processing techniques of dual-energy CT enabled distinct attenuation ratio ranges to be defined for uric acid, cystine, struvite, calcium oxalate, calcium phosphate, and brushite stones [14].

It has been shown that NCCT collimation width comparable to or larger than the size of the measured stone results in volume-averaging effects with the surround, resulting in an underestimation of the true HU [5]. This is supported by the current study, particularly for stones < 10 mm as seen in Fig. 1. A strength of this study is the overall large size of calculi in the series. Furthermore, to minimise volume averaging error the protocol mandated selection of the largest stone in each patient and drawing a circular region of interest at the centre of the stone, thus avoiding edge artefact. We concede that the largest calculus may not be representative of all calculi in an individual, and different regions within the same stone have different attenuation which has been averaged in this study.

The single patient in our series with HU > 1000 had multiple historical treatments for cystinuria in another centre, but none in the last 7 years. The NCCT reviewed in this series demonstrated bilateral calculi. The patient is on preventative urinary alkinisation treatment with potassium citrate. It is well known that high urine pH associated with alkalinisation of the urine can lead to calcium phosphate stone formation and conversion from cystine to non-cystine stones has been demonstrated in 25% of patients [15]. Stone analysis from previous PCNL showed 90% cystine and 10% Calcium phosphate (apatite) in this patient, thus the high HU in this patient likely represents the mixed nature of this stone. A limitation of our study is that the calculus composition was not available for all patients for further evaluation, and more patients in the series may have stones of mixed composition.

**Fig. 2:**
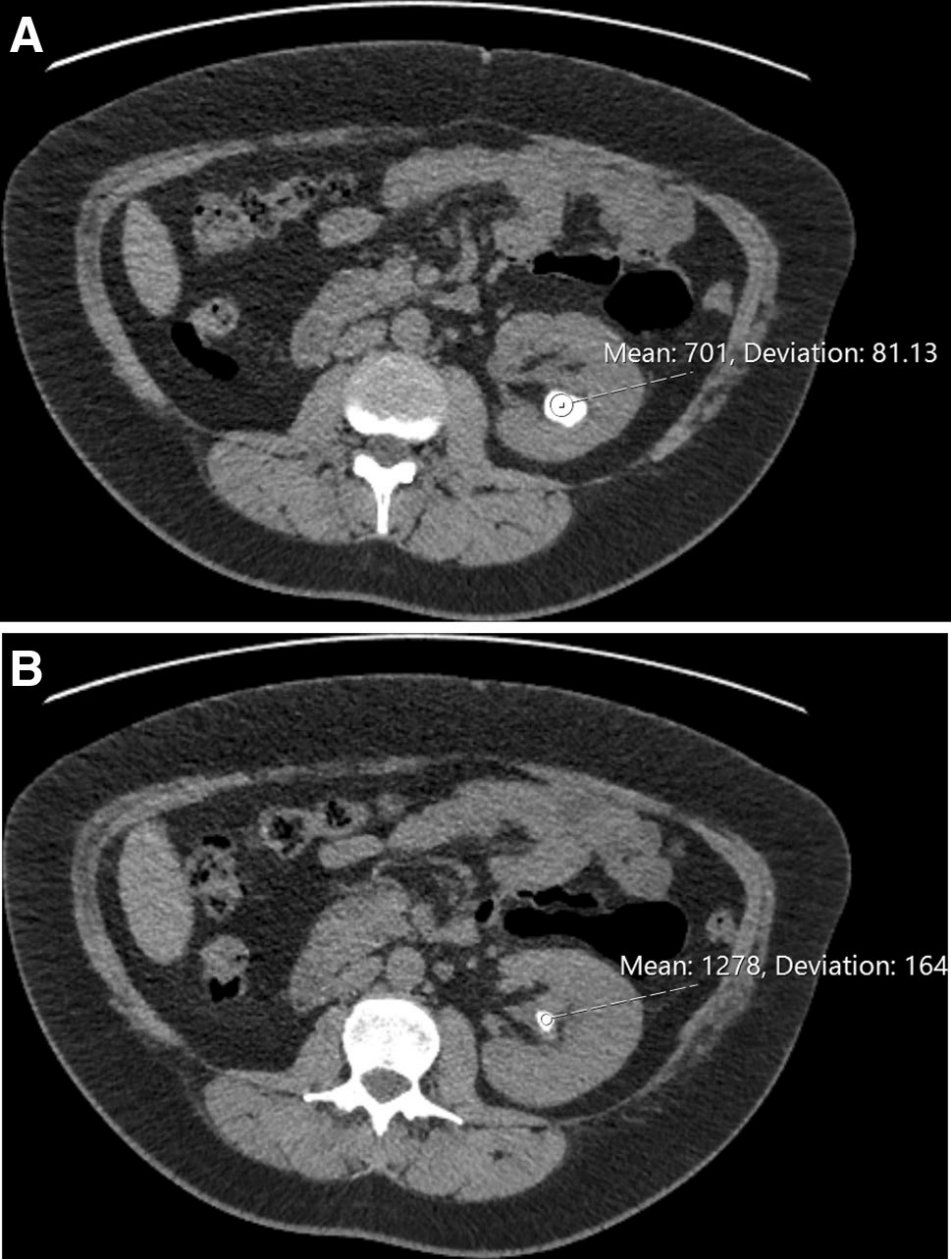
Partial staghorn stone. The majority of the stone had a HU measurement of 701 (Fig. 2a) whilst the more central component had a HU measurement of 1278 (Fig. 2b)

**Fig. 3:**
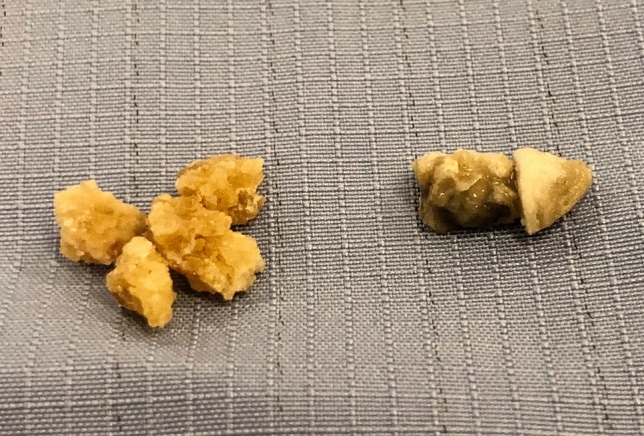
Stone extracted at time of PCNL. The stone on the left was from the area in Fig. 2a and has the typical appearance of cystine. This was predominantly cystine on analysis. The stone on the right (from the area in Fig. 2b) has different appearance and was calcium phosphate on analysis without a cystine component

In another example of how HU measurement might be useful clinically, one patient presented with recurrent stone on follow-up. On CT scans the stone showed two distinct areas with HU of 701 and 1278 (Fig. 2). At PCNL the appearance of these two areas was very different (Fig. 3) and stone analysis demonstrated predominantly cystine from the first area and calcium phosphate from the second.

Cystine stones have previously been described as distinct ‘rough’ or ‘smooth’ entities, where ‘rough’ stones have lower attenuation values [9] and are more susceptible to treatment [16] than ‘smooth’ stones. These two studies utilised micro CT in vitro to demonstrate the internal structure of 31 and 26 cystine stones, respectively. Furthermore, Patel et al. demonstrated two distinct groups of HU < 550 and HU > 850 in a series of 20 cystinuria patients in vivo [17]. All measurements were performed by a single scanner, with consistent energy and reconstruction settings [17]. In our study, this phenomenon was not observed. We acknowledge this may be due to the real-world nature of our series with inconsistent scanner models and settings [18]. We therefore suggest it is not yet possible to determine the internal ‘rough’ or ‘smooth’ structure of cystine stones in vivo in the routine clinical setting.

## Conclusion

The current study demonstrates that mean HU measured in vivo was low for stones that are often deemed difficult to treat. Cystinuria genotype was not associated with mean HU. Furthermore, HU cannot yet reliably differentiate rough and smooth cystine stones in vivo, and therefore cannot be used to identify stones susceptible to extracorporeal shockwave lithotripsy. Future generations of CT scanners may make this possible, and help avoid numerous instrumentations of the urinary tract in this patient population, of particular importance given their rate of stone formation. From our data, when assessing patients with unknown stone composition, a HU of < 800 should prompt consideration of cystinuria, while HU > 1000 are very unlikely to be pure cystine. Furthermore, a change from low to high HU in a patient with cystinuria should raise the possibility of non-cystine stone formation.

## Electronic supplementary material

Below is the link to the electronic supplementary material.

Supplementary file1 (xlsx 57 KB)
